# Possible influence of *Plasmodium*/*Trypanosoma* co-infections on the vectorial capacity of *Anopheles* mosquitoes

**DOI:** 10.1186/s13104-020-04977-8

**Published:** 2020-03-04

**Authors:** Maty Fofana, Christian Mitri, Diawo Diallo, Brice Rotureau, Cheikh Tidiane Diagne, Alioune Gaye, Yamar Ba, Constentin Dieme, Mawlouth Diallo, Ibrahima Dia

**Affiliations:** 1 Pôle de Zoologie Médicale, Institut Pasteur de Dakar, 36 Avenue Pasteur, BP 220, Dakar, Sénégal; 2 Unité Génétique et Génomique des Insectes Vecteurs, Institut Pasteur, Paris, France; 3 Trypanosome Transmission Group, Trypanosome Cell Biology Unit, INSERM U1201 & Department of Parasites and Insect Vectors, Institut Pasteur, Paris, France; 4 Wadsworth Center, New York State Department of Health, Slingerlands, NY USA

**Keywords:** *Trypanosoma*, *Plasmodium*, Mixed infection, *Anopheles*, Senegal

## Abstract

**Objective:**

In tropical Africa, trypanosomiasis is present in endemic areas with many other diseases including malaria. Because malaria vectors become more anthropo-zoophilic under the current insecticide pressure, they may be exposed to trypanosome parasites. By collecting mosquitoes in six study sites with distinct malaria infection prevalence and blood sample from cattle, we tried to assess the influence of malaria-trypanosomiasis co-endemicity on the vectorial capacity of *Anopheles*.

**Results:**

Overall, all animal infections were due to *Trypanosoma vivax* (infection rates from 2.6 to 10.5%) in villages where the lowest *Plasmodium* prevalence were observed at the beginning of the study. *An. gambiae* s.l. displayed trophic preferences for human-animal hosts. Over 84 mosquitoes, only one was infected by *Plasmodium falciparum* (infection rate: 4.5%) in a site that displayed the highest prevalence at the beginning of the study. Thus, *Anopheles* could be exposed to *Trypanosoma* when they feed on infected animals. No *Plasmodium* infection was observed in the *Trypanosoma*-infected animals sites. This can be due to an interaction between both parasites as observed in mice and highlights the need of further studies considering *Trypanosoma*/*Plasmodium* mixed infections to better characterize the role of these infections in the dynamic of malaria transmission and the mechanisms involved.

## Introduction

Malaria and African Trypanosomiases also known as Human African Trypanosomiasis (HAT) or sleeping sickness in humans and Animal African Trypanosomiasis (AAT) or Nagana in animals, are endemic diseases in tropical Africa [1–3]. They are respectively caused by protists of the genera *Plasmodium* and *Trypanosoma* that are transmitted to humans and animals by the infective bites of the blood-feeding insect vectors of the genera *Anopheles* and *Glossina,* respectively. In many parts of tropical Africa, sleeping sickness occurs in areas where malaria is endemic [1]. Although published reports of co-infections are scarce [4–6], *Anopheles* and *Glossina* vectors can theoretically feed on hosts carrying *Plasmodium* and/or *Trypanosoma,* and thus become exposed simultaneously or consecutively to these parasites. We reasonned that such a co-exposure may impact on the development of *Plasmodium* in *Anopheles* vectors and consequently on its transmission.

In Senegal, although no HAT case has been reported for at least one decade [7, 8], AAT remains endemic, mainly in the Kédougou region. The last survey from 1971 to assess the distribution of the disease, reported the presence of *T. congolense* and *T. brucei* in cattle, horses, donkeys and carnivores, and the presence of *T. vivax* infections was reported in cattle and horses [9]. As in many African countries, malaria is still a public health problem in Senegal. Although valuable efforts were undertaken to reduce its burden, the Kédougou area remains one with the highest incidence of malaria [10]. Indeed, the ecology of the region is favourable to the maintenance of high seasonal malaria transmission with entomological inoculation rates raising up to 200 infective bites and *Plasmodium* incidence rates greater than 25 per 1000 inhabitants [11].

In a recent study, Sanches-Vaz and others [12] showed through a co-infection model, that mice primarily infected with *T. brucei*, followed by the administration of *P. berghei* sporozoites, are protected from experimental cerebral malaria and presented increased host survival. In addition, subsequent infection of *An. coluzzii* in mice infected with *Trypanosoma brucei brucei* and *Plasmodium yoelii* showed a significant impact of infection as compared to mosquitoes fed on *Plasmodium yoelii* mono-infected mice [13]. These observations raise the question of the possible impact of malaria-trypanosomiasis co-endemicity on the *Plasmodium* transmission by *Anopheles* in the field.

The present study was conducted in the Kédougou region where both malaria and trypanosomiasis are endemic in six study sites of this region selected for their distinct malaria infection prevalence. The objective was to update the last epidemiological data on AAT published more than 40 years ago and further investigate the possible link of *Anopheles* exposure to *Trypanosoma* on their vectorial capacity for the malaria parasite *Plasmodium*.

## Main text

### Methods

#### Selection of the study sites

**Fig. 1:**
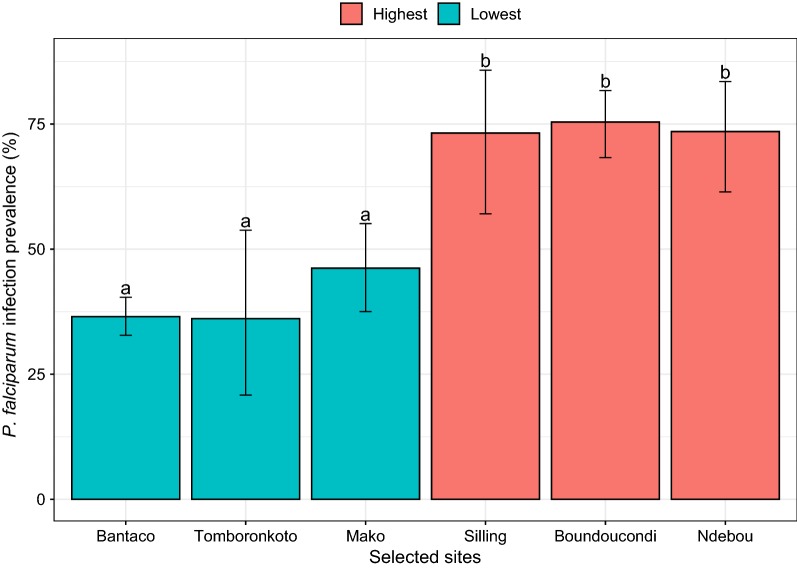
Variations of *P. falciparum* infection rates among the outpatients received in the health centers in the Kédougou area. The bars indicate the upper 95% confidence interval associated to the prevalence. Prevalences with different letters are significantly different (p < 0.05)

The study was conducted in the Kédougou region where malaria and AAT co-circulate. This area is situated in a transition zone between the dry tropical forest and the savannah belt. There is one rainy season that lasts from June to November. Mean temperatures vary from 33 to 39.5 °C during the year. The population is predominantly rural and is estimated at 141,226 inhabitants with 55% of people under 20 years [14, 15]. The average population density is estimated at 8 inhabitants per km^2^, mostly living in small-scattered villages. The incidence of malaria in this region is among the highest in Senegal (15 per 1000 inhabitants) with the highest prevalence of *P. falciparum* (14%) among children under 5 years [10]. For trypanosomiases, no human case has been recorded for at least one decade. However, AAT is known to be endemic and only a specific cattle race (trypanotolerant Ndama) is adapted in the area. *T. vivax, T. congolense* and, in a lesser extent, *T. brucei* are also recorded in neighbouring countries. Based on the data available from 48 different health centers within the Kédougou region [15], six villages were purposively selected based on *P. falciparum* prevalence infection outcomes from outpatients (Fig. 1, Additional file 1). Mako, Bantaco and Tomboronkoto exhibited the lowest *Plasmodium* prevalence (respectively 46.2%, 36.5% and 36.1%) while Silling, Ndébou and Boundoucondi displayed the highest *Plasmodium* prevalence (respectively 73.2%, 73.5% and 75.4%).

#### Blood sample collection in animals

At the beginning of the study in October 2015, before mosquito collection, blood was collected from cattle (bovines, sheeps and goats) in each of the six sites, into a vacuum tube containing EDTA. After collection, the tubes were kept at room temperature for at least 2 h and the serum was recovered after centrifugation and stored at − 80 °C in the laboratory.

#### Mosquito collections

Adult mosquitoes were collected using two sampling methods: (1) CDC miniature light traps hung next to a sleeper under untreated bed nets indoor and outdoor from 7:00 pm to 7:00 am during three consecutive nights; (2) Indoor PSC in October 2015, October 2016 and December 2016 in ten bedrooms in each of the six sites.

Upon collection, mosquitoes were sorted and identified morphologically following the key of Gillies and de Meillon [16]. Bloodmeals from fed specimens collected by PSC were blotted onto a filter paper for host source identification in the laboratory. All mosquitoes were stored individually in numbered tubes with desiccant for laboratory processing.

#### Laboratory processing

The origin of blood meals from fully blood fed mosquito females was identified as human, bovine, ovine, sheep and horse using the ELISA from the procedure of Beier et al. [17].

Females from the *Anopheles gambiae* complex were identified at the species level using the method of Fanello et al. [18].

*Plasmodium* and *Trypanosoma* infections were detected using respectively the nested-PCR of Snounou et al. [19] and the ITS1 “Touchdown” PCR from the procedure of Tran et al. [20].

#### Data analysis

For *Plasmodium* and *Trypanosoma* infection, the infection rates were calculated as the proportion of mosquito specimens or animals found positive by PCR. The anthropophilic/zoophilic rates were calculated as the proportion of human/animal blood to the total blood meals identified by ELISA.

For statistical analysis, Chi square and Fisher exact tests were used with P values < 0.05 considered as significant.

### Results

#### Mosquito collections

Overall, 252 anopheline specimens were collected during the three sampling sessions. *An. gambiae* s.l. was the predominant species in each of the six villages with a mean frequency of 75% (range 53.8–100%). It was followed by *An. funestus* (13%), *An. rufipes* (6%), *An. flavicosta* (2%), *An. squamosus* (2%), *An. nili* (1.2%), *An. domicola* and *An. pharoensis* (0.4% each).

Within the *An. gambiae* complex, *An. arabiensis* was the predominant species representing 60.4% followed by *An. gambiae* (37.9%) and *An. coluzzii* (1.7%).

#### Trophic preferences

A total of 191 blood meals from *An. gambiae* females were tested by ELISA. Overall, 44% of the identified blood meals originated from single blood meals (either from human or animal sources). The others were represented by mixed blood meals either from human and animal hosts (34.8%) or from two animal hosts (21.2%).

At the site level, *An. gambiae* were seen to be anthropo-zoophagic in all villages, excepted in Ndébou where they were highly anthropophilic (Table 1).

**Table 1:** Trophic preferences of *An. gambiae* females from the 6 sites visited

Sites	Tested	Single blood meals		Mixed blood meals		Undet.
		Human	Animal	HA	AA	
Bantaco	8	3 (37.5%)	3 (37.5%)	2 (25%)	0 (0%)	1 (11.1%)
Tomboronkoto	17	10 (58.8%)	1 (5.9%)	2 (11.8%)	4 (23.5%)	0 (0%)
Mako	60	11 (18.3%)	14 (23.3%)	22 (36.7%)	13 (21.7%)	1 (1.6%)
Boundoucondi	46	13 (28.3%)	6 (13%)	17 (37%)	10 (20%)	4 (8%)
Ndébou	16	15 (93.8%)	0 (0%)	0 (0%)	1 (6.3%)	0 (0%)
Silling	37	1 (2.7%)	4 (10.8%)	21 (56.8%)	11 (29.7%)	0 (0%)

*HA* human-animal, *AA* animal–animal, *Undet.* undetermined

#### *Plasmodium* and *Trypanosoma* infections

In *An. gambiae* s.l. populations, no infection neither by *Trypanosoma* nor by *Plasmodium* parasite was detected by PCR in mosquitoes collected in 2015. However, from a total of 84 specimens collected in 2016, one *An. arabiensis* from the village of Boundoucondi was found harbouring *Plasmodium falciparum* (Table 2). The estimated *P. falciparum* infection rate was 4.5% (95% CI 0.1–22.8%).

**Table 2:** *P. falciparum* and *T. vivax* infection rates respectively in *An. gambiae* females and animals in the different villages visited

Sites	*An. gambiae* s.l.			Animals		
	*P. falciparum*			*T. vivax*		
	Tested	Positive	%	Tested	Positive	%
Bantaco	7	0	0	55	5	9.1 (3–19.9)
Tomboronkoto	11	0	0	38	4	10.5 (2.9–24.8)
Mako	24	0	0	38	1	2.6 (0.1–13.8)
Ndébou	15	0	0	47	0	0
Silling	5	0	0	57	0	0
Boundoucondi	22	1	4.5 (0.1–22.8)	49	0	0

Values in brackets indicate the 95% confidence interval

Regarding the infectious status of the animals, a total of 284 sera were tested by PCR for *Trypanosoma* detection. Among positive samples, only *Trypanosoma vivax* was detected in 10 animals from 3 different villages (Table 2). The trypanosome infection rates were estimated respectively to 9.1% (95% CI 3–19.9%) in Bantaco, 10.5% (95% CI 2.9–24.8%) in Tomboronkoto and 2.6% (95% CI 0.1–13.8%) in Mako. These villages presented the lowest *Plasmodium* infection rates at the beginning of the study. In Bantaco and Mako, all the infected animals were goats, whereas in Tomboronkoto, three sheeps and one goat were found infected. The animal infection rates were significantly different between the 6 villages (Fisher exact test, p = 0.005).

### Discussion

The objective of this study was to update the epidemiological profile of AAT in South-East Senegal and to investigate a possible link between *Trypanosoma* infection and *Anopheles* vectorial capacities for malaria.

The presence of *Trypanosoma* was investigated in domestic animals from each of the 6 selected villages. Our results showed the presence of *T. vivax* in ten animals out of 284 in Bantaco, Mako and Tomboronkoto; the three sites that showed the lowest *Plasmodium* infections in human at the beginning of the study. In these sites, no *Plasmodium* infection was observed in mosquitoes. Several studies conducted in the area have shown the presence of trypanotolerant animals [9, 21–23]. The detection of *T. vivax* in animals from the Kédougou region confirms the data already obtained by Touré [9] who showed that this species was mainly found in cattle and horses, not only in this area but also across all the country and also the presence of *T. congolense* and *T. brucei* in cattle, horses, donkeys and carnivores in tsetse-infested areas. A more recent cross-sectional study conducted between June 2011 and September 2012 in Dakar, Sine Saloum, Kédougou and Casamance has revealed the presence of *T. congolense* and *T. brucei* in dogs, donkeys, horses, cattle, sheep, goats with an average infection rate of 3.4% [24]. Concerning *T. vivax*, only one goat from the village of Dielmo was found infected.

The entomological findings showed the predominance of *An. arabiensis* exhibiting variables trophic preferences with mixed blood meals from both human and domestic animals as well as from several animals. *Trypanosoma* parasites were not detected in *Anopheles* mosquitoes. These observations were made from mosquitoes freshly fed on animals or both human and animals. The lack of detection of *Trypanosoma* from mosquito could possibly be due to the short lag of time that the parasite can survive in its organism. Indeed, during their studies Dieme et al, (personal communication) have found that *Trypanosoma* parasites can only survive in the mosquito midgut for about 24 h after blood feeding.

*P. falciparum* was detected in the village of Boundoucondi (from 1 *An. arabiensis*) which presented, with Ndébou and Silling, the highest *P. falciparum* infection rates at the beginning of our study. While infected mosquitoes were observed only in the village of Boundoucondi, the estimated infection rate in this village was higher than that observed by Dia et al, [25] in the area. The absence of *P. falciparum*-infected mosquitoes in the majority of village could be due to the low observed anthropophilic rates combined to the relative important numbers of mixed blood meals on human-animal and animal–animal vertebrate hosts. These results indicate, however, that *Anopheles* mosquitoes could be exposed to *Trypanosoma* parasites when they feed on infected animals. Thus, as observed by Sanches-Vaz and others [12] in mice, this can conduct to a reduction of infection in *Anopheles* mosquitoes. Indeed, the absence of plasmodial infection in the sites where animals were found infected with *T. vivax* (sites with low malaria prevalence) and the presence of plasmodial infections in sites without trypanosome-infected animals, would be in favour of the involvement of trypanosomal infection in the plasmodial infection.

This study confirms the presence of AAT in the South-East region of Senegal and provides indications that *Anopheles* could be exposed to *Trypanosoma* parasites when they feed on infected animals. It highlights the need of further studies taking into account *Trypanosoma* and *Plasmodium* mixed infections to better characterize the role of these infections in the dynamic of malaria transmission and the mechanisms involved.

## Limitations

This work is not an comprehensive and thorough mechanistic study of the interaction of the two parasites and cannot therefore provide a deeper insight into the mechanisms involved. It shows however that *Anopheles* mosquitoes could be exposed to *Trypanosoma* parasites when they feed on infected animals that in turn could reduce *Plasmodium* infection. We would like to investigate this assumption through a study of the impact of *Trypanosoma* infection in the dynamic of malaria transmission and the mechanisms involved experimentally using articial co-infection with trypanosomes and gametocytes.

## Supplementary information

**Additional file 1.** Localisation of the selected villages in the Kédougou region. Red and yellow colors denote respectively the villages with highest and lowest *P. falciparum* prevalence. The size of the bubble is proportional to the observed prevalences. This map was built using a shapefile from the free domain of the Geographic Information System (http://www.diva-gis.org) with the R software version 3.3.1 and the package rgdal.

## Data Availability

All data generated or analysed during this study are included in this manuscript.

## Abbreviations

CDC: Centers for Disease Control
PCR: Polymerase chain reaction
HAT: Human African Trypanosomiasis
AAT: Animal African Trypanosomiasis
EDTA: Ethylene diamine tetraacetic acid
PSC: Pyrethrum spray collection
ELISA: Enzyme-Linked Immunosorbent Assay
ITS: Internal transcribed spacer
CI: Confidence interval
