# Supplementary figures and images for: Possible influence of Plasmodium/Trypanosoma co-infections on the vectorial capacity of Anopheles mosquitoes
Source: BMC Res Notes. 2020 Mar 4;13:127. doi: 10.1186/s13104-020-04977-8 (PMC7057563; doi:10.1186/s13104-020-04977-8)

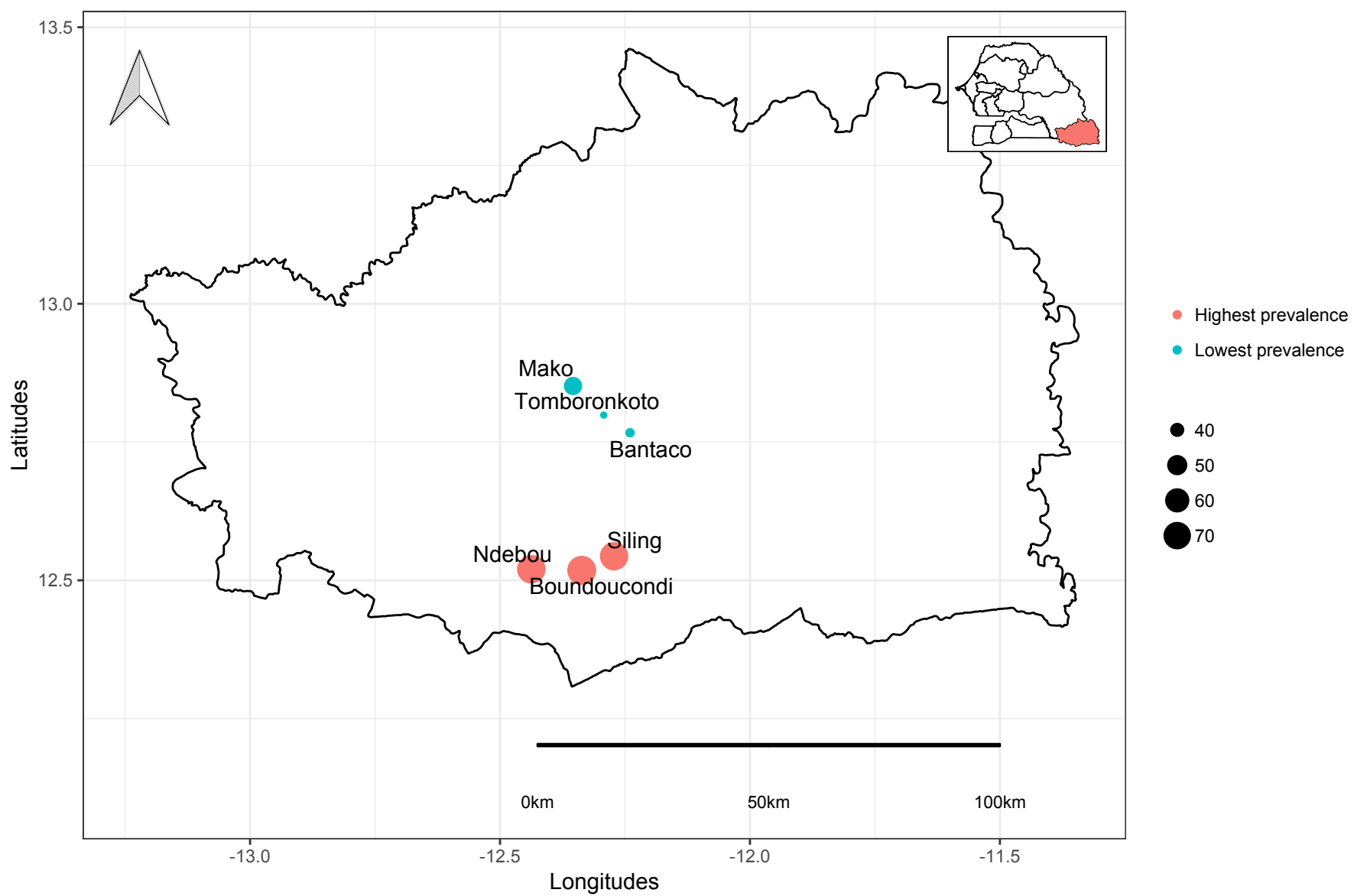

Supplement: Supplementary file 1 — Additional file 1. Localisation of the selected villages in the Kédougou region. Red and yellow colors denote respectively the villages with highest and lowest P. falciparum prevalence. The size of the bubble is proportional to the observed prevalences. This map was built using a shapefile from the free domain of the Geographic Information System (http://www.diva-gis.org) with the R software version 3.3.1 and the package rgdal. [file 13104_2020_4977_MOESM1_ESM.pdf]
